# Singing at 0.1 Hz as a Resonance Frequency Intervention to Reduce Cardiovascular Stress Reactivity?

**DOI:** 10.3389/fpsyt.2022.876344

**Published:** 2022-04-27

**Authors:** Sandra Tanzmeister, Christian Rominger, Bernhard Weber, Josef M. Tatschl, Andreas R. Schwerdtfeger

**Affiliations:** Institute of Psychology, University of Graz, Graz, Austria

**Keywords:** cardiovascular resonance, coherent breathing, heart rate variability, mental stress, resonance breathing

## Abstract

Slow breathing at 6 breaths per min (corresponding to ~ 0.1 Hz) has been found to benefit psychological and physical health. In this study, we aimed to examine if paced singing at 0.1 Hz has beneficial acute effects on physiological function as compared to slow breathing. Participants were randomized to one of four experimental interventions prior to performing a mental stress task: paced breathing at 0.1 Hz (*n* = 26), paced singing at 0.1 Hz (*n* = 26), spontaneous breathing (*n* = 24), or spontaneous singing (*n* = 25). Heart rate, heart rate variability in the low (LF-HRV) and high frequency (HF-HRV) domain, blood pressure and affective wellbeing were assessed. As expected, both paced breathing and paced singing resulted in elevated LF-HRV. Moreover, both singing groups evidenced increases in heart rate, blood pressure and positive affect, thus indicating elevated sympathetic activation. Breathing and singing at 0.1 Hz had no robust effect on cardiovascular stress reactivity. Findings suggest that paced singing could constitute a promising alternative to slow paced breathing as it increases cardiovascular coherence, although more studies are needed to elucidate whether slow breathing and/or singing could ameliorate acute stress responses.

## Introduction

Breathing at around 6 breaths per min (corresponding to 0.1 Hz) has been found to evoke coherent oscillations in various physiological systems, which has been referred to as resonance frequency meaning maximal coherent oscillations with substantial benefits for bodily function [e.g., (1)]. Of note, slow breathing strongly amplifies the variability of cardiac beat to beat-intervals [so-called heart rate variability, HRV; (2)]. Specifically, the link between heart rate (HR) and breathing is indicated by respiratory sinus arrhythmia (RSA), which describes the phenomenon that causes HR to increase during inhalation and decrease during exhalation, ultimately increasing HRV. During resting conditions, healthy adults exhibit breathing frequencies between 9 and 20 breaths per min, which corresponds to 0.15–0.33 Hz [see e.g., (3)]. A lower breathing frequency causes RSA to shift from the high frequency (HF = 0.15–0.4 Hz) to the low frequency (LF = 0.04–0.15 Hz) domain (4, 5), which is mainly due to vagal influences (6). Hence, breathing at 0.1 Hz leads to a vagally-mediated increase in spectral power in the LF domain, which has been related to baroreceptor reflex sensitivity [e.g., (7)]. Of note, such a kind of slow breathing has been suggested to benefit health and psychological wellbeing [e.g., (8–10)]. Consequently, a slowed breathing rate has been considered a treatment strategy for stress-related diseases and autonomic nervous system dysfunction (4, 6, 11, 12), depression [e.g., (13)], and anxiety and perceived stress [e.g., (14)].

There are various methods to produce a breathing frequency of 0.1 Hz, like yogic breathing, during which a person inhales for 4 s and exhales for 6 s (15), or diaphragmatic breathing, where the abdomen expands while the chest stays relatively low (1). Other methods involve specific voice-related activity, like reciting the rosary prayer. Bernardi et al. (16), for example, have shown that reciting one cycle of the rosary prayer lasts exactly 10 s (0.1 Hz). In this respect, singing could also be particularly useful to produce the breathing frequency of 0.1 Hz (11, 17). Vickhoff et al. (17), for example, composed a melody which evoked a breathing rhythm of exactly 10 s corresponding to 6 breaths per min. Specifically, participants were instructed to sing according to the repetitive song structure (with a tempo of 48 bpm) of three subsequent half notes and one half-pause (at which participants had time to breathe). Importantly, this song resulted in a clear LF-HRV power increase.

In addition to the beneficial effects of slow breathing, singing (in groups and solo) was found to go along with beneficial physiological effects, such as decreases of cortisol and increases in immune function, thus suggesting lower stress and improved immunocompetence (18–22). Additionally, singing seems to improve feelings of social connectedness, the sense of self and subjective wellbeing (23). This is in line with the finding that positive emotions resulting from singing could mediate the stress-ameliorating effects of singing, while in the absence of those positive emotions the stress-reducing effect of singing appears to be lowered (19, 20). Hence, singing under individually pleasant circumstances could be a useful tool to buffer stress.

It should be noted that, to the authors' knowledge, studies analyzing the effects of 0.1 Hz breathing or singing on acute cardiovascular reactivity to mental stress are limited to date. Previous studies, for example, suggested that slow breathing may dampen the psychophysiological response to anticipated threat (24, 25). Moreover, Whited et al. (26) found that a 0.1 Hz biofeedback training enduring 4–8 weeks had a rather fragile HRV-enhancing effect during stress in the treatment as compared to the control group and Chin and Kales (27) also reported elevated HRV during a mild cognitive stress task as a result of a single 5 min-slow breathing exercise. Finally, Steffen et al. (28) could observe that a single session of 0.1 Hz breathing training resulted in attenuated systolic blood pressure (SBP) to a mental stress task and recovery period. However, most of the previous studies must be considered underpowered and selective with respect to the variables reported (either HRV or blood pressure).

Based on Vickhoff et al.'s (17) findings that singing at 0.1 Hz could have beneficial physiological effects potentially stimulating vagal efference, this study aimed to examine if singing in combination with slow paced breathing is associated with a more adaptive response to stress. Specifically, we hypothesized that, first, singing at 0.1 Hz and breathing at 0.1 Hz would result in an increase in LF-HRV as compared to the spontaneous (unregulated) groups. We also hypothesized that positive affect would increase more substantially in the singing as compared to the breathing groups. Second, since singing as well as paced breathing have been suggested to have salutary organismic effects [e.g., (16, 19, 29, 30)], we hypothesized that the combined effect of singing and slow breathing (i.e., singing at breathing rate of 0.1 Hz) would result in a lower cardiovascular stress reactivity than spontaneous singing or slow breathing alone. In order to evaluate the presumed adaptive effect of singing in combination with slow breathing in more detail, we established four randomized experimental interventions with a respective duration of 5 min: paced singing at 0.1 Hz, paced breathing at 0.1 Hz, spontaneous singing, and spontaneous breathing. The participating experienced singers completed one of the interventions before being faced with a mental stress task.

## Methods

### Participants

An a priori power analysis was conducted to calculate the required sample size. According to Steffen et al. (28), we aimed to detect a medium-sized-interaction effect (*f* = 0.25) at a significance level of 0.05 with a power of 0.80. Specifying a two-way ANOVA with the independent factors time of measurement (baseline, intervention, stressor and recovery) and intervention (0.1 Hz breathing, 0.1 Hz singing, spontaneous breathing and spontaneous singing) a sample size of *N* = 100 was required. We recruited 106 participants, of whom three had to be excluded from further analysis due to suspicion of hypertension at baseline measurement (blood pressure > 149/90 mmHg) and two because of excessive artifacts (one for blood pressure artifacts and one for ECG artifacts), which might have distorted the results of the statistical analyses. Thus, the study comprised of 101 healthy amateur singers (79 women, 22 men) aged 18 to 44 (*M* = 25.43, *SD* = 6.21) with a mean waist to hip-ratio of 0.75 (*SD* = 0.07). Participants were recruited from several Styrian choirs, ensembles, music conservatories, music schools and music universities and hence, were either members of an amateur choir, amateur ensemble or singers of an amateur band. Exclusion criteria included professional singers, cardiovascular diseases, diabetes, psychiatric disorders and pregnancy, as these variables could have influenced cardiovascular activity. The research was approved by the local ethics committee (GZ. 39/61/63 ex 2018/2019). Informed consent was obtained from all participants prior to study entry.

### Study Design and Experimental Manipulation

**Figure 1:**
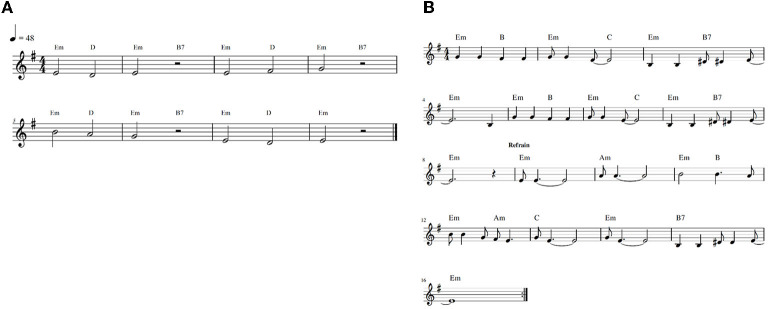
**(A)** Paced singing melody. **(B)** “Go Down Moses” (spontaneous singing).

Participants were randomly assigned to one of four experimental interventions. In this phase, the experimental task lasted 5 min. For intervention (1) *paced breathing at 0.1 Hz* (PB; *n* = 26), participants were asked to inhale for 4 s and exhale for 6 s indicated by a time bar, to produce a breathing rhythm of 0.1 Hz. For intervention (2) *paced singing at 0.1 Hz* (PS; *n* = 26), participants were asked to sing a simple, short song in a loop (see, Figure 1A). This song structure was based on the song structure that Vickhoff et al. (17) used in their study. The tempo was 48 bpm, which means that two bars lasted exactly 10 s. Thus, when singing three half notes (which equals exhaling) without pause and only breathing at the indicated half pause (which equals inhaling), participants inhaled 4 s and exhaled for 6 s. In (3) *spontaneous breathing* (SB; *n* = 24), participants were listening to an excerpt of the audio book “The Little Prince”. During (4) *spontaneous singing* (SS; *n* = 25), participants were asked to sing the melody of the song “Go Down Moses” (tempo: 120 bpm) in a loop (see Figure 1B). This song had no fixed breathing pattern. To avoid any influence of verbal information, participants in the PS and SS interventions were asked to sing both melodies without text, but with syllables of their choice (e.g., “do do”). Both PS and SS were accompanied by a previously recorded piano melody. For interventions 1–3 there was a short training period before the actual intervention began, so that participants could become familiar to the breathing rhythm and/or the songs, respectively. Written instructions and acoustic stimuli were delivered *via* computer screen and loudspeakers.

### Stress Task

We used the well-established serial subtraction task as a mental challenge [e.g., (31, 32)]. In this task, participants are asked to mentally calculate serial subtractions and report the results at each step to the experimenter (i.e., subtract 13 from 6,233, then subtracting 13 from 6,220 and so on). Participants were not allowed to use any auxiliary means and were instructed to calculate as fast as possible. If they miscalculated, they had to restart from 6,233. To increase stress, participants were told beforehand that they would be filmed during the whole task and that their performance would be evaluated and compared to others. This phase of the experiment lasted 5 min.

### Variables and Instruments

#### Affective State Assessment

Positive and negative affect (PA, NA) were assessed with the German version of the *Positive and Negative Affect Schedule* [PANAS; (33)]. This schedule includes 10 adjectives to describe PA (e.g., happy, active) and 10 for NA (e.g., angry, nervous). Across study phases, reliability (Cronbach's Alpha) for PA ranged between 0.77 and 0.92 and for NA between 0.72 and 0.86, thus suggesting reliable assessment.

#### Physiological Measurements

Physiological signals were recorded continuously throughout the entire experimental session and task periods were defined using digital triggers. HR was measured by means of an ECG device (AccuSync^®^ 72, Milford, Connecticut, USA) using a modified Einthoven II-point lead. The ECG was recorded using Ambu BlueSensor^®^ electrodes (Ballerup Sogn, Denmark) with a sampling rate of 1,000 Hz. The signal was recorded with the software AcqKnowledge^®^ 4.3 (Biopac Systems Inc., Goleta, California, USA). HR and HRV were analyzed offline with Kubios premium software [vers. 3.2; University of Finland (34)], thereby applying artifact correction if necessary. LF-HRV and HF-HRV as a sensitive indicator of vagally-mediated HRV [e.g., (4, 35)] were analyzed. HRV variables were log-transformed prior to analysis to account for skewness.

Continuous blood pressure (SBP; diastolic blood pressure, DBP) was measured by non-invasive measurement of arterial finger BP using the Finometer^®^ PRO (Finapres Medical Systems, Amsterdam). The signal was recorded using the software BeatScope^®^ Easy (v2.10). After visual inspection, mean SBP and DBP were calculated for each task period for each participant. Table 1 shows *M* and *SD* for all interventions and all physiological variables.

**Table 1:** Means and standard deviation of all cardiovascular variables for the factors of experimental intervention and time.

		**0.1 Hz breathing**		**0.1 Hz singing**		**Spontaneous breathing**		**Spontaneous singing**	
		***n*** **=** **26**		***n*** **=** **26**		***n*** **=** **24**		***n*** **=** **25**	
		***M***	***SD***	***M***	***SD***	***M***	***SD***	***M***	***SD***
SBP (mm/Hg)	Baseline	126.24	9.53	125.14	8.33	128.60	9.31	132.34	12.88
	Intervention	124.31	10.28	140.44	12.55	130.65	9.68	141.29	14.54
	Stressor	148.10	16.16	151.64	14.63	156.53	18.80	149.66	17.99
	Recovery	133.10	13.04	134.19	11.66	139.34	13.16	138.42	15.18
DBP (mm/Hg)	Baseline	74.36	6.55	73.64	6.36	76.51	6.54	77.71	8.32
	Intervention	73.25	6.51	84.07	8.52	77.84	6.80	87.86	8.98
	Stressor	88.84	9.29	91.50	9.71	94.29	10.60	92.05	10.10
	Recovery	79.00	8.63	79.67	7.54	83.01	8.95	83.73	9.07
HR (BPM)	Baseline	72.48	11.58	74.12	9.63	74.78	9.07	73.89	11.56
	Intervention	75.96	10.89	81.51	9.44	74.57	8.85	84.28	9.32
	Stressor	82.32	10.11	89.46	14.56	88.98	11.03	85.28	12.30
	Recovery	71.13	10.06	74.16	9.05	73.62	8.77	71.46	9.72
Log HF-HRV (ms^2^)	Baseline	6.67	1.41	6.82	1.16	6.48	1.16	6.77	1.38
	Intervention	6.65	1.31	6.70	0.95	6.26	1.04	6.68	0.77
	Stressor	6.44	1.16	6.15	1.17	6.54	0.66	6.39	0.76
	Recovery	6.55	1.48	6.70	1.09	6.53	1.12	6.80	1.31
Log LF-HRV (ms^2^)	Baseline	6.71	1.29	7.04	1.17	6.78	0.94	7.01	0.98
	Intervention	8.95	0.78	8.63	0.73	6.68	0.66	7.11	0.62
	Stressor	7.42	0.81	7.24	0.91	7.48	0.68	7.37	0.46
	Recovery	7.01	1.32	7.11	1.07	6.88	0.86	7.11	0.79

*SBP, systolic blood pressure; DBP, diastolic blood pressure; HR, heart rate; HF-HRV, high frequency heart rate variability; LF-HRV, low frequency heart rate variability*.

### Procedure

**Figure 2:**
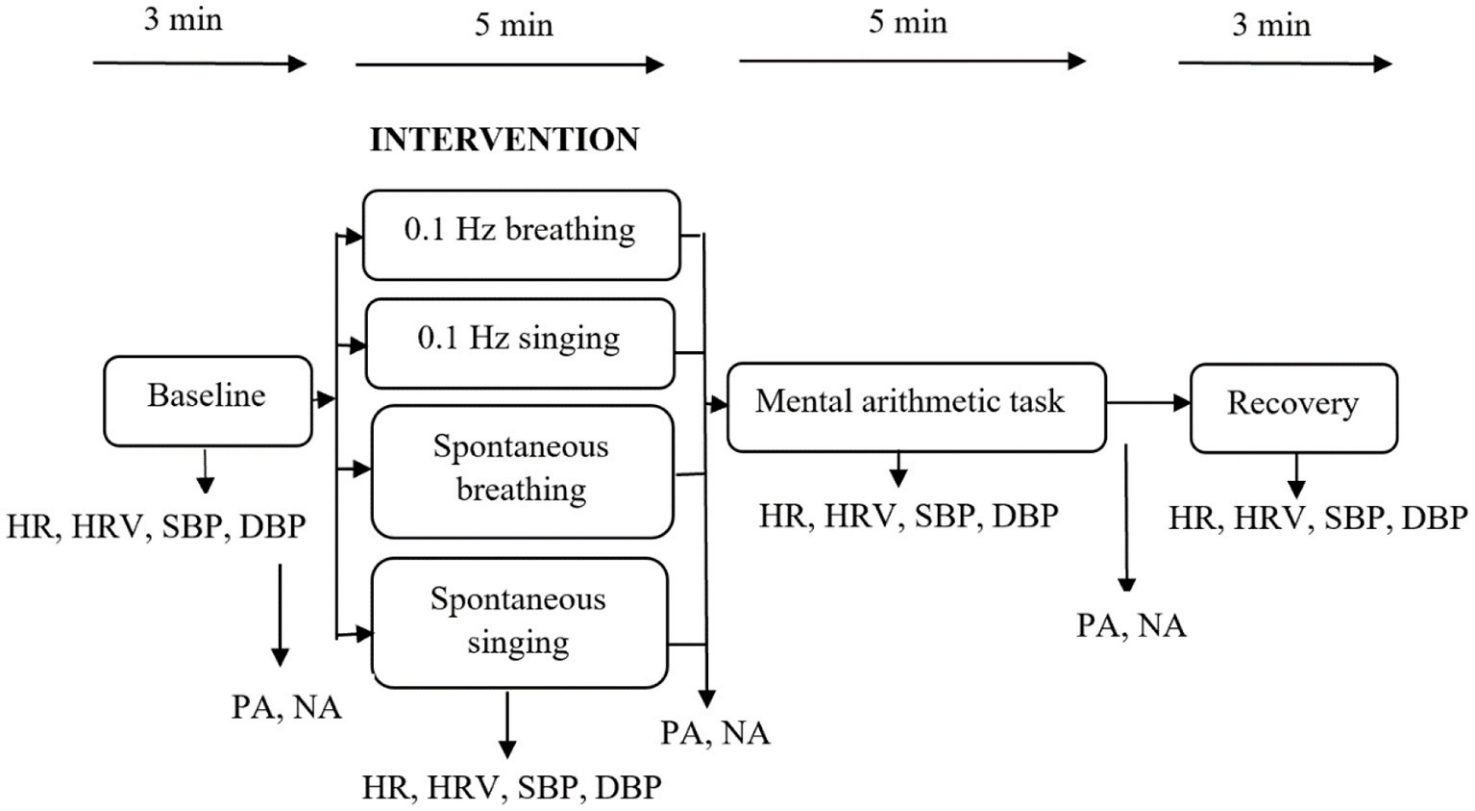
Study procedure and main variables. HR, heart rate; HRV, heart rate variability; SBP, systolic blood pressure; DBP, diastolic blood pressure; PA, positive affect; NA, negative affect.

Upon arrival, participants received informational pages on the study and signed informed consent. Afterwards, their height, weight and abdominal circumference were measured. The physiological sensors were attached and participants were randomly assigned to one of the four experimental interventions. Randomization was accomplished by appearence at the laboratory, irrespective of age and sex. Severe unbalance in the course of the study was monitored and resolved if necessary. The physiological assessment started with a 3-min baseline recording, during which participants were shown landscape photographs. Subsequently, they completed the PANAS. Afterwards they underwent one of the four experimental interventions for a period of 5 min, followed by a second PANAS assessment. Then, the mental arithmetic task was conducted followed by a third PANAS assessment. Subsequently, a recovery period of 3 min was implemented. Throughout the whole experiment, HR, SBP and DBP were recorded. Figure 2 gives an overview of the study procedure.

### Statistical Analysis

In order to examine the effects of the intervention, five 2-way mixed analyses of variance (ANOVAs) were calculated for between subjects' data for each of four levels (paced breathing, paced singing, spontaneous breathing and spontaneous singing) and the within subjects' data over time (at baseline and at intervention). Hypotheses regarding stress reactivity were further analyzed *via* 2-way mixed ANOVAs with intervention (4 groups) and time (baseline, stressor and recovery) as factors. HR, LF-HRV, HF-HRV, SBP, and DBP served as the main dependent variables. Moreover, a 4 (intervention) by 3 (baseline, intervention, stress) ANOVA was conducted for PA and NA, respectively, to track differences in affective wellbeing. *Post-hoc* analyses were carried out using Tukey's honest significant difference (HSD) *post-hoc* tests. Two-tailed significance testing was performed at *p* < 0.05. Degrees of freedom were corrected when necessary, using the Greenhouse-Geisser correction.

## Results

### Intervention Effects

#### LF-HRV

**Figure 3:**
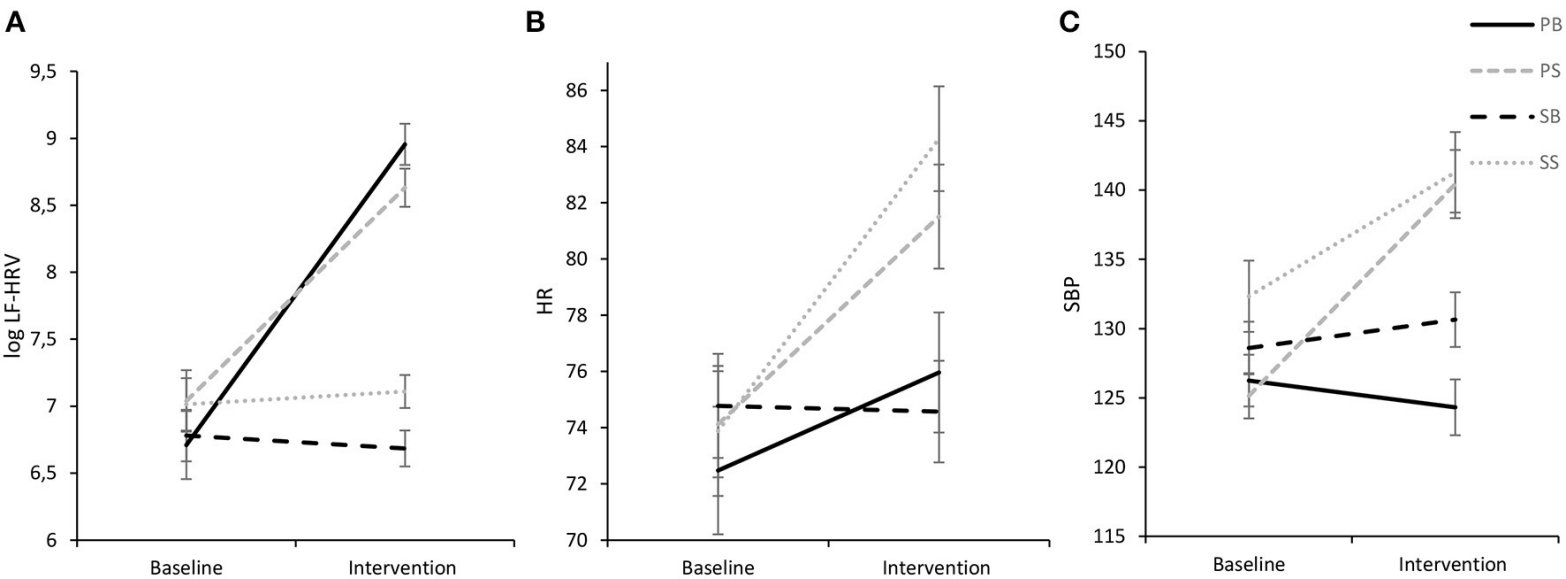
Experimental intervention effects for log LF-HRV **(A)**, HR **(B)**, and SBP **(C)** (M ± 1 SE). log LF-HRV, low-frequency HRV; HR, heart rate; SBP, systolic blood pressure; ln, natural logarithmic transformation. PB, paced breathing at 0.1 Hz; PS, paced singing at 0.1 Hz; SB, spontaneous breathing; SS, spontaneous singing. This figure show elevated LF-HRV for both paced interventions and increases in cardiovascular activity for both singing interventions.

Analysis of between-group data reached significance [*F*_(3,97)_ = 11.67, *p* < 0.001, η_*p*_^2^ = 0.27], as did within-subject data over time [*F*_(1,97)_ = 118.09, *p* < 0.001, η_*p*_^2^ = 0.55], respectively, which however, were further qualified by a significant intervention by time interaction [*F*_(3,97)_ = 41.86, *p* < 0.001, η_*p*_^2^ = 0.56], indicating a large effect. Pairwise comparisons showed that LF-HRV increased significantly from baseline to paced singing (*p* < 0.001; Cohen's *d* = 1.66) and paced breathing (*p* < 0.001; Cohen's *d* = 2.81), respectively, with large effect sizes. Conversely, LF-HRV did not change significantly from baseline to both spontaneous singing (*p* = 0.595; Cohen's *d* = 0.11) and spontaneous breathing (*p* = 0.600; Cohen's *d* = 0.11). Furthermore, a Tukey-HSD *post-hoc* test showed that while interventions did not differ significantly from each other during baseline, LF-HRV was significantly higher during intervention in the paced interventions in comparison to the spontaneous interventions (*ps* < 0.001). LF-HRV during paced breathing and paced singing did not differ significantly from each other (*p* = 0.099), while LF-HRV during spontaneous breathing was significantly lower than during spontaneous singing (*p* = 0.036). Results are visualized in Figure 3A.

#### HF-HRV

Analysis of HF-HRV indicated no significant main effects of intervention [*F*_(3,97)_ = 0.57, *p* = 0.572] and time [*F*_(1,97)_ = 1.41, *p* = 0.237], and no significant interaction of time and intervention [*F*_(3,97)_ = 0.18, *p* = 0.909].

#### HR

Analysis of HR indicated a significant main effect of time [*F*_(1,97)_ = 137.52, *p* < 0.001, η_*p*_^2^ = 0.59], but no significant main effect of intervention [*F*_(3,97)_ = 1.46, *p* = 0.229, η_*p*_^2^ = 0.04]. However, the main effect of time was further qualified by a significant interaction of time and intervention [*F*_(3,97)_ = 25.80, *p* < 0.001, η_*p*_^2^ = 0.44] with a Tukey-HSD *post-hoc* test documenting that HR did not differ significantly between the interventions during baseline. However, during the intervention the spontaneous singing intervention evidenced a higher HR than both the spontaneous breathing intervention (*p* = 0.001) and the paced breathing intervention (*p* = 0.003). Moreover, the paced singing intervention evidenced a higher HR than both the spontaneous breathing intervention (*p* = 0.013) and the paced breathing intervention (*p* = 0.041). Pairwise comparisons revealed that HR significantly increased from baseline to paced singing (*p* < 0.001, *d* = 1.23), spontaneous singing (*p* < 0.001, *d* = 1.98) and paced breathing (*p* < 0.001, *d* = 0.97), respectively, but did not change for spontaneous breathing (*p* = 0.307; *d* = 0.10). Results are displayed in Figure 3B.

#### SBP and DBP

ANOVAs for SBP and DBP revealed significant intrasubject data over time [SBP: *F*_(1,97)_ = 72.15, *p* < 0.001, η_*p*_^2^ = 0.43; DBP: *F*_(1,97)_ = 139.98, *p* < 0.001, η_*p*_^2^ = 0.59] and between-subjects data for intervention [SBP: *F*_(3,97)_ = 5.54, *p* = 0.001, η_*p*_^2^ = 0.15, DBP: *F*_(3,97)_ = 7.12, *p* < 0.001, η_*p*_^2^ = 0.18], which however, were further validated by significant interactions of time and intervention for both SBP [*F*_(3,97)_ = 28.73, *p* < 0.001] and DBP [*F*_(3,97)_ = 46.46, *p* < 0.001] with large effect sizes each (SBP: η_*p*_^2^ = 0.47; DBP: η_*p*_^2^ = 0.59).

As SBP and DBP showed very similar findings, only data for SBP will be reported in the following. A Tukey-HSD *post-hoc* test showed that while SBP did not differ significantly between interventions during baseline, during intervention SBP in both singing interventions was significantly higher than SBP in both breathing interventions (*p*'s ≤ 0.005) as is illustrated in Figure 3C. Both breathing interventions and both singing interventions did not differ significantly from each other (breathing interventions: *p* = 0.064, singing interventions: *p* = 0.800). Pairwise comparisons further showed that SBP increased significantly from baseline to paced singing (*p* < 0.001, *d* = 1.48), spontaneous singing (*p* < 0.001, *d* = 1.09) and spontaneous breathing (*p* = 0.016, *d* = 0.53), respectively. Noteworthy, SBP decreased significantly from baseline to paced breathing (*p* = 0.023, *d* = 0.47), indicating a medium-sized effect.

### Cardiovascular Stress Reactivity

#### LF-HRV

**Figure 4:**
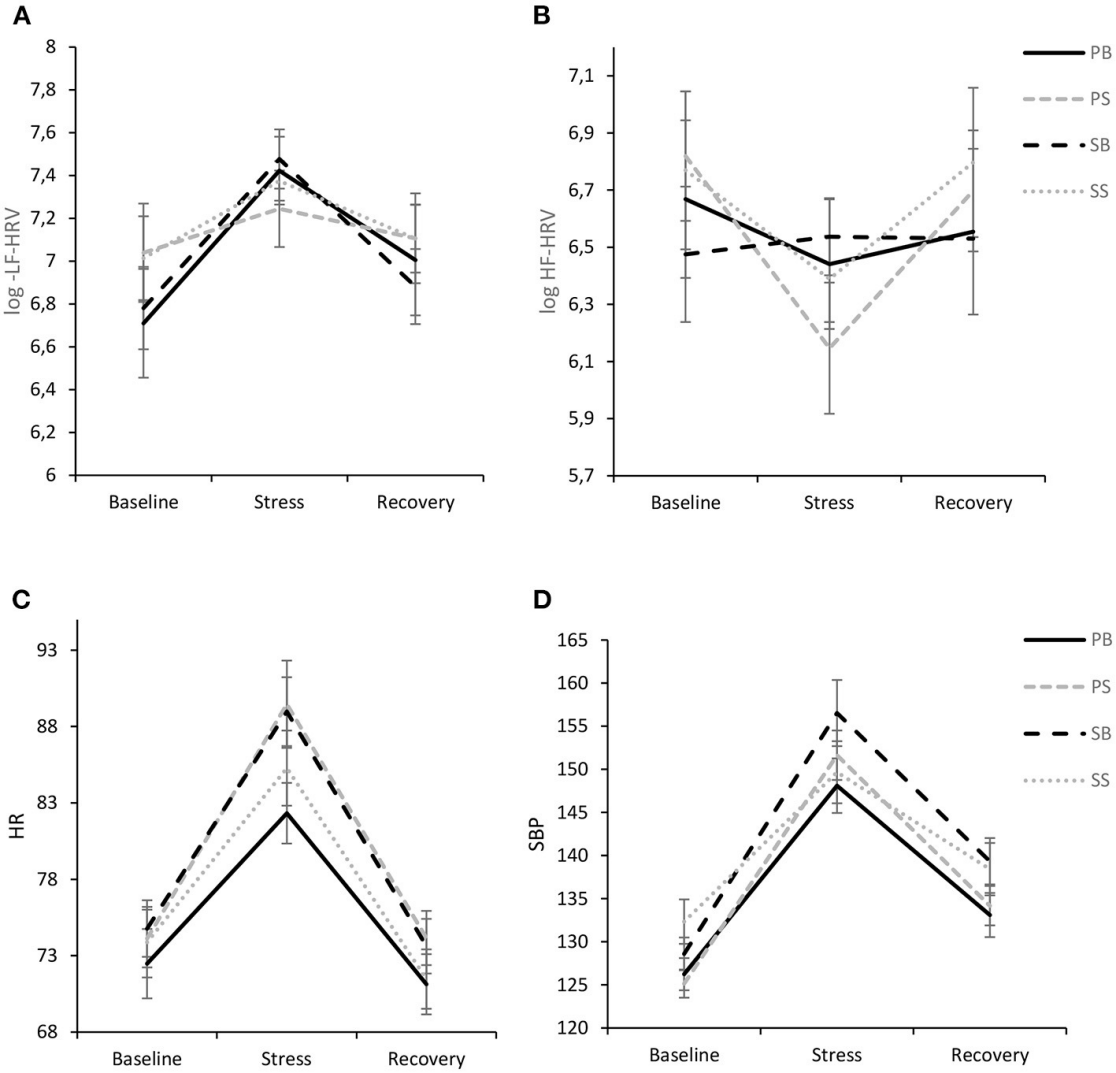
Interaction of experimental intervention and time (M ± 1 SE) for log LF-HRV **(A)**, log HF-HRV **(B)**, HR **(C)**, and SBP **(D)**. PB, paced breathing at 0.1 Hz; PS, paced singing at 0.1 Hz; SB, spontaneous breathing; SS, spontaneous singing. This figure show pronounced cardiovascular stress reactivity, but no differences between interventions.

Analysis of LF-HRV only revealed a significant intrasubject effect of time [*F*_(1.725, 167.369)_ = 14.46, *p* < 0.001, η_*p*_^2^ = 0.13] with pairwise comparisons indicating that LF-HRV significantly increased from baseline to the stress task (*p* < 0.001, *d* = 0.45). The reduction of LF-HRV from stress to recovery was also significant (*p* < 0.001, *d* = 0.36). Between-subjects effect of intervention did not reach significance [*F*_(3,97)_ = 0.14, *p* = 0.936, η_*p*_^2^ = 0.004] and there was no significant interaction of time and intervention [*F*_(5.176, 167.369)_ = 1.06, *p* = 0.388, η_*p*_^2^ = 0.03]. Results are visualized in Figure 4A.

#### HF-HRV

For HF-HRV there was a significant intrasubject effect of time [*F*_(1.671, 162.065)_ = 7.58, *p* < 0.001, η_*p*_^2^ = 0.07], while there was no between-subjects effect of intervention [*F*_(3,97)_ = 0.07, *p* = 0.974] and no intervention by time interaction [*F*_(5.012, 162.065)_ = 1.92, *p* = 0.093, η_*p*_^2^ = 0.06], although a trend toward a more pronounced vagal withdrawal to stress was evident in the paced singing intervention with a subsequent rebound (Figure 4B).

#### HR

The ANOVA revealed only a significant intrasubject effect of time [*F*_(1.230, 119.353)_ = 191.73, *p* < 0.001, η_*p*_^2^ = 0.66] with pairwise comparisons showing that HR was significantly higher during the stressor in comparison to baseline (*p* < 0.001, *d* = 1.28) and recovery (*p* < 0.001, *d* = 1.59), respectively. Furthermore, HR was significantly lower during recovery in comparison to baseline (*p* = 0.002, *d* = 0.31). No other effects were significant (see Figure 4C). In particular, there was no significant time by intervention interaction [*F*_(3.691, 119.353)_ = 1.53, *p* = 0.203, η_*p*_^2^ = 0.05].

#### SBP and DBP

The ANOVA for SBP revealed a significant intrasubject effect of time [*F*_(1.599, 155.086)_ = 262.02, *p* < 0.001, η_*p*_^2^ = 0.73], which was further qualified by a significant interaction of time and group [*F*_(4.796, 155.086)_ = 2.74, *p* = 0.023], indicating a medium effect size (η_*p*_^2^ = 0.08). In general, all groups evidenced a stress response with significant increases from baseline to stressor with large effect sizes (all *p*'s <0.001; PS: *d* = 2.50, PB: *d* = 1.89, SS: *d* = 1.15, SB: *d* = 2.06) and a significant decline from stressor to recovery (PS: *d* = 1.96, PB: *d* = 1.81, SS: *d* = 1.25, SB: *d* = 1.57). In general, recovery values exceeded baseline values with medium to large-sized effects (PS: *d* = 1.01, PB: *d* = 0.81, SS: *d* = 0.59, SB: *d* = 1.58). Of note, the spontaneous singing group showed a significantly lower response than the other groups and particularly the paced singing group [*F*_(1.623, 79.546)_ = 4.91, *p* = 0.015, η_*p*_^2^ = 0.09], which became evident by decomposing the two-way interaction between time and group. Results are visualized in Figure 4D.

For DBP there was a significant intrasubject effect of time [*F*_(1.597, 154.873)_ = 427.33, *p* < 0.001, η_*p*_^2^ = 0.82] and no significant interaction of time and intervention [*F*_(4.790, 154.873)_ = 1.87, *p* = 0.106, η_*p*_^2^ = 0.06] as well as no between-subjects effect of intervention [*F*_(3,97)_ = 1.58, *p* = 0.199, η_*p*_^2^ = 0.05].

### State Affect in the Course of the Experiment

**Figure 5:**
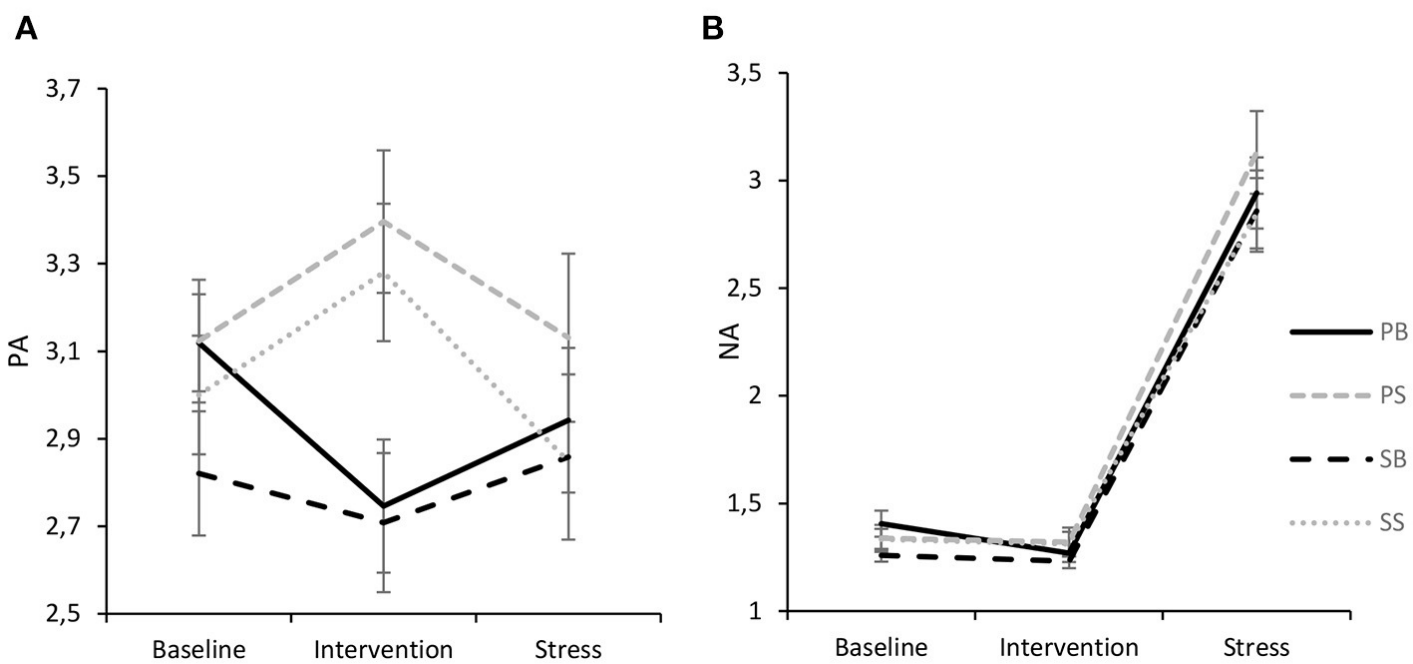
Interaction of experimental intervention and time (M ± 1 SE) for PA **(A)** and NA **(B)**. PB, paced breathing at 0.1 Hz; PS, paced singing at 0.1 Hz; SB, spontaneous breathing; SS, spontaneous singing. It is shown that both singing interventions increased PA, while paced breathing led to a deterioration of PA. NA only evidenced a pronounced stress effect.

For PA (Figure 5A), the ANOVA revealed no significant intrasubject effect of time [*F*_(1.796, 174.198)_ = 1.23, *p* = 0.291, η_*p*_^2^ = 0.01] and no between-subject effect of intervention [*F*_(3,97)_ = 0.85, *p* = 0.470, η_*p*_^2^ = 0.03], respectively, while a significant interaction of time and intervention [*F*_(5.388, 174.198)_ = 4.77, *p* < 0.001] indicating a medium effect size (η_*p*_^2^ = 0.13) was found. Tukey-HSD *post-hoc* tests further showed that while interventions did not differ during baseline (*p* = 0.678) and stress (*p* = 0.849), respectively, PA was significantly higher following paced singing than both paced breathing (*p* = 0.010) and spontaneous breathing (*p* = 0.019). Likewise, following spontaneous singing, PA was significantly higher than after spontaneous breathing (*p* = 0.027) and paced breathing (*p* = 0.015), respectively. Pairwise comparisons showed that PA increased significantly from baseline to spontaneous singing, indicating a medium-sized effect (*p* = 0.012, *d* = 0.55). For paced singing, a similar increase could be observed (*p* = 0.009, *d* = 0.50). On the contrary, paced breathing led to a significant decrease in PA (*p* = 0.003, *d* = 0.64) while spontaneous breathing was not associated with a reliable change from baseline (*p* = 0.192, *d* = 0.27). Moreover, a significant reduction in PA from intervention to stress could be observed for spontaneous singing (*p* < 0.001, *d* = 0.79), while the reduction was not reliable for the other interventions (*p*s > 0.05).

For NA (Figure 5B), there was no significant interaction effect of time and intervention [*F*_(3.356, 108.495)_ = 0.18, *p* = 0.924, η_*p*_^2^ = 0.006] and no significant between-subject effect of intervention [*F*_(3,970)_ = 0.74, *p* = 0.529, η_*p*_^2^ = 0.02]. However, a significant intrasubject effect of time [*F*_(1.119, 108.495)_ = 270.34, *p* < 0.001, η_*p*_^2^ = 0.74] indicated that NA marginally significantly decreased from baseline to intervention (*p* = 0.060, *d* = 0.19) and significantly increased from intervention to stressor (*p* < 0.001, *d* = 1.71). Finally, after the stressor NA was significantly higher than after baseline (*p* < 0.001, *d* = 1.67). It should be noted though that NA levels in general were rather low.

## Discussion

The main aim of this study was to investigate the effects of slow singing and breathing at 0.1 Hz on cardiovascular function and to evaluate their respective impact on cardiovascular stress reactivity in a sample of experienced singers. First, it turned out that paced singing at 0.1 Hz resulted in a similar increase in LF-HRV to slow breathing. However, it also provoked comparably stronger increases in SBP, HR and PA, while 0.1 Hz breathing led to a significant decrease of blood pressure. Second and unexpectantly, spontaneous singing resulted in a lower SBP stress reactivity as compared to singing at 0.1 Hz, while there were no reliable intervention differences for the other cardiovascular variables.

### Both Paced Singing and Paced Breathing Increased Resonance Frequency

Research suggests that vagal activity during slow breathing (respiratory frequency of 0.1 Hz) can produce RSA oscillations that extend into the LF frequency band of HRV (36). Our findings confirm that singing at 0.1 Hz provoked similar changes in RSA as 0.1 Hz breathing. In particular, while HF-HRV did not change from baseline to intervention, LF-HRV strongly increased with large effect sizes. This pattern of finding generally indicates that the manipulation of breathing produced positive results in both paced interventions, thus suggesting that singing at 0.1 Hz could be considered a similarly powerful tool to evoke resonance frequency and thus, to stimulate baroreceptor function (7). Hence, this finding further supports previous research (11, 16, 17), which suggests that 0.1 Hz singing, humming or chanting mantras that follow a specific musical structure could constitute suitable alternatives to 0.1 Hz breathing for inducing cardiovascular resonance [e.g., (29)]. Other previous research found a similar elevated HRV pattern during slow singing without words, in low pitch and not requiring effort (37).

It should be noted that LF-HRV during 0.1 Hz breathing has been attributed to vagal origin (6), thus suggesting a parasympathetic stimulation. Importantly, while LF-HRV was similarly enhanced in individuals performing paced singing and paced breathing, HR and SBP differentiated between interventions. In particular, singing also induced substantial increases in blood pressure and HR, while 0.1 Hz breathing reduced blood pressure. While the decrease in blood pressure in the latter intervention could indicate the effect of baroreceptor stimulation, the reason for the blood-pressure increasing effect during 0.1 Hz singing could be attributed to enhanced bodily (muscles) activation by producing sounds in comparison to (soundless) breathing. Importantly, Lehrer et al. (7) showed that rhythmic muscle contractions paced at 0.1 Hz resulted in elevated LF-HRV and pronounced SBP oscillations. Of note, the marked increase in both HR and blood pressure in addition to the strong increase in RSA during 0.1 Hz singing might reflect a combined influence of both sympathetic and parasympathetic nerve fibers. In contrast, higher HR and blood pressure during spontaneous singing without alterations in HRV are compatible with the assumption of a stronger sympathetic efference. Although the health effect of a more general activation of both branches of the autonomic nervous system during paced singing remains to be elucidated in future research, it should be noted that it was also associated with higher wellbeing (elevated PA), which may argue for a generally beneficial activation pattern.

### No Reliable Acute Effects of 0.1 Hz Singing or Breathing on Cardiovascular Stress Reactivity

Our findings could not confirm a robust ameliorating effect of 0.1 Hz singing or breathing on cardiovascular reactivity to mental stress. Conversely, spontaneous singing led to a lower SBP reactivity as compared to 0.1 Hz singing. It could be speculated that the combination of slow breathing and singing might have increased burden as it may represent a dual task. However, also breathing alone at 0.1 Hz did not result in a reliable modification of the cardiovascular stress response. Hence, this study deviates from previous research suggesting attenuated physiological stress reactivity of 0.1 Hz breathing [e.g., (24–28)]. For example, Whited et al. (26) found evidence for a mild stress buffering role of slow breathing on HRV and Steffen et al. (28) reported attenuated SBP reactivity and recovery resulting from slow breathing. It should be noted though that Steffen et al. (28) used a longer training phase of 15 min, while the individual resonance frequency for each person was determined. Whited et al. (26) even implemented a 5- to 8-week slow (biofeedback) breathing training with 30 min each week. Hence, it could be speculated that the 5-min intervention in the present study (in individuals not previously familiar with this kind of paced breathing) was insufficient to reliably modulate cardiovascular stress responses. It should also be noted that some of the previous studies applied rather mild stress tasks that did either not reliably induce stress responses (28), found reliable effects only for a particular cardiac measure [pNN50; (26)], or examined rather small samples in each intervention seldom exceeding *n* = 12, thus challenging the robustness of the findings [e.g., (24, 25, 27)]. Nonetheless, it could be speculated that the pacing during singing and breathing could have been problematic in that it might have provoked additional demands on the participants, thus undermining a general stress-relieving effect.

### Singing Raises PA

Previous research (20, 21) showed that singing improves wellbeing. We found that singing increased PA for both paced and unpaced singing with medium effect sizes. Noteworthy, PA remained rather the same after spontaneous breathing and even decreased significantly after paced breathing. It must be noted though that while singing resulted in elevated PA, this effect was rather short-lived as PA after the stress task decreased to the same level in all four interventions. However, it should be kept in mind that participants sung for only 5 min and that an increase in PA could be found even after this short time period, although in almost all other studies that explored this effect (19–21), participants engaged in singing for at least half an hour. Importantly, the findings suggest that due to the stimulating effect of singing on PA, combining slow breathing with singing could be beneficial for ensuring participants' compliance during long-term breathing interventions to benefit health.

### Limitations of the Study

Although this study provides support for acute coherence enhancing effects of combining 0.1 Hz breathing with singing, some limitations should be discussed. First, in the literature the impact of singing on mood or physiological stress indicators has usually been measured in the context of a rehearsal lasting at least half an hour (21). In the present study, however, individuals sang/breathed for only 5 min, so it may well be that the positive physical and psychological responses usually elicited by singing and/or 0.1 Hz breathing could not have been elicited to the full extent. Future research should thus strive for longer intervention periods and/or a higher dosage. In this respect, there is evidence that professional singers might particularly benefit from the physiological effects of singing [i.e., exhibit a particularly pronounced increase of LF-HRV; (38)], which further suggests that a more extensive singing engagement could prove particularly positive for health. Second, the study sample was quite homogeneous, consisting mainly of academics (ungraduated and graduated students). Moreover, the sample was composed of hobby singers (who may have been very positive about singing), since otherwise random assignment to the interventions would have been impossible or very difficult (possibly imposing a stress induction due to singing for many naïve individuals). Interestingly, Grape et al. (38) could also show that amateur singers were particularly enthusiastic when engaging in singing. Hence, it needs to be evaluated in future research if the beneficial physiological and psychological effects of 0.1 Hz singing and breathing, respectively, can be generalized to individuals without singing experience. Finally, although both sexes were examined, men were underrepresented, thus precluding generalizability of the results.

## Conclusion

Our study confirms a growing body of scientific research on the immediate positive psychological as well as physiological effects of (slow) singing. Specifically, by analyzing cardiovascular activity and subjective affect throughout the study period, we found that paced singing at 0.1 Hz was associated with a similarly elevated LF-HRV like 0.1 Hz breathing, which is in accordance with studies suggesting vagally stimulating effects of slow breathing, humming or singing. Moreover, higher HR, SBP and PA resulted during both 0.1 Hz singing and free singing, which confirms the activating effect of singing in general. Although it has been suggested that slow paced breathing could counteract cardiovascular disease [such as chronic hypertension (16, 39, 40)] and generally benefit physical and mental health [e.g., (2, 8, 10, 36)], acute effects of a brief intervention on cardiovascular reactivity could not be supported. More studies are certainly needed in order to examine dosage-response effects in more detail.

## Data Availability Statement

The raw data supporting the conclusions of this article will be made available by the authors, without undue reservation.

## Ethics Statement

The studies involving human participants were reviewed and approved by University of Graz Local Ethics Committee (GZ. 39/61/63 ex 2018/2019). The patients/participants provided their written informed consent to participate in this study.

## Author Contributions

ST: study conceptualization, manuscript writing, and data analysis. CR: study conceptualization, data parametrization, study implementation, and manuscript writing. BW: study implementation, study realization, and paradigm programming. JMT: data visualization, manuscript writing, and data parametrization. ARS: manuscript writing, study funding, manuscript writing, and statistical analysis. All authors contributed to the article and approved the submitted version.

## Conflict of Interest

The authors declare that the research was conducted in the absence of any commercial or financial relationships that could be construed as a potential conflict of interest.

## Publisher's Note

All claims expressed in this article are solely those of the authors and do not necessarily represent those of their affiliated organizations, or those of the publisher, the editors and the reviewers. Any product that may be evaluated in this article, or claim that may be made by its manufacturer, is not guaranteed or endorsed by the publisher.

## References

[1] Russo MA Santarelli DM O'Rourke D. The physiological effects of slow breathing in the healthy human. Breathe. (2017) 13:298–309. 10.1183/20734735.009817 29209423 PMC5709795

[2] Schwerdtfeger AR Schwarz G Pfurtscheller K Thayer JF Jarczok MN Pfurtscheller G. Heart rate variability (HRV): from brain death to resonance breathing at 6 breaths per minute. Clin Neurophysiol. (2020) 131:676–93. 10.1016/j.clinph.2019.11.013 31978852

[3] Berntson GG Bigger JT Eckberg DL Grossman P Kaufmann PG Malik M. Heart rate variability: origins, methods, and interpretive caveats. Psychophysiology. (1997) 34:623–48. 10.1111/j.1469-8986.1997.tb02140.x 9401419

[4] Shaffer F McCraty R Zerr CL. A healthy heart is not a metronome: an integrative review of the heart's anatomy and heart rate variability. Front Psychol. (2014) 5:1040. 10.3389/fpsyg.2014.01040 25324790 PMC4179748

[5] Lohninger A editor. Herzratenvariabilität: Das HRV-Praxis-Lehrbuch. Wien: Facultas (2017). p. 423.

[6] Kromenacker BW Sanova AA Marcus FI Allen JJ Lane RD. Vagal mediation of low-frequency heart rate variability during slow yogic breathing. Psychosom Med. (2018) 80:581–7. 10.1097/PSY.0000000000000603 29771730

[7] Lehrer PM Vaschillo E Trost Z France CR. Effects of rhythmical muscle tension at 01Hz on cardiovascular resonance and the baroreflex. Biol Psychol. (2009) 81:24–30. 10.1016/j.biopsycho.2009.01.003 19428965

[8] Mather M Thayer J. How heart rate variability affects emotion regulation brain networks. Curr Opin Beha Sci. (2018) 19:98–104. 10.1016/j.cobeha.2017.12.017 29333483 PMC5761738

[9] Lehrer PM Gevirtz R. Heart rate variability biofeedback: how and why does it work? Front Psychol. (2014) 5:756. 10.3389/fpsyg.2014.00756 25101026 PMC4104929

[10] Lehrer PM Kaur K Sharma A Shah K Huseby R Bhavsar J. Heart rate variability biofeedback improves emotional and physical health and performance: a systematic review and meta analysis. Appl Psychophysiol Biofeedback. (2020) 45:109–29. 10.1007/s10484-020-09466-z 32385728

[11] Bossinger W. Die heilende Kraft des Singens: Von den Ursprüngen bis zu modernen Erkenntnissen über die soziale und gesundheitsfördernde Wirkung von Gesang. Norderstedt: Books on Demand (2005). p. 296.

[12] Song H-S Lehrer PM. The effects of specific respiratory rates on heart rate and heart rate variability. Appl Psychophysiol Biofeedback. (2003) 28:13–23. 10.1023/a:1022312815649 12737093

[13] Tatschl JM Hochfellner SM Schwerdtfeger AR. Implementing mobile HRV biofeedback as adjunctive therapy during inpatient psychiatric rehabilitation facilitates recovery of depressive symptoms and enhances autonomic functioning short-term: a 1-year pre–post-intervention follow-up pilot study. Front Neurosci. (2020) 14:e00738. 10.3389/fnins.2020.00738 32792897 PMC7386054

[14] Goessl VC Curtiss JE Hofmann SG. The effect of heart rate variability biofeedback training on stress and anxiety: a meta-analysis. Psychol Med. (2017) 47:2578–86. 10.1017/S0033291717001003 28478782

[15] Pramanik T Sharma HO Mishra S Mishra A Prajapati R Singh S. Immediate effect of slow pace bhastrika pranayama on blood pressure and heart rate. J Altern Complement Med. (2009) 15:293–5. 10.1089/acm.2008.0440 19249921

[16] Bernardi L Sleight P Bandinelli G Cencetti S Fattorini L Wdowczyc-Szulc J. Effect of rosary prayer and yoga mantras on autonomic cardiovascular rhythms: comparative study. BMJ. (2001) 323:1446–9. 10.1136/bmj.323.7327.1446 11751348 PMC61046

[17] Vickhoff B Malmgren H Aström R Nyberg G Ekström S-R Engwall M. Music structure determines heart rate variability of singers. Front Psychol. (2013) 4:334. 10.3389/fpsyg.2013.00334 23847555 PMC3705176

[18] Beck RJ Cesario TC Yousefi A Enamoto H. Choral singing, performance perception, and immune system changes in salivary immunoglobulin A and cortisol. Music Percept. (2000) 18:87–106. 10.2307/40285902

[19] Beck RJ Gottfried TL Hall DJ Cisler CA Bozeman KW. Supporting the health of college solo singers: the relationship of positive emotions and stress to changes in salivary IgA and cortisol during singing. J Learning Arts. (2006) 1:19. 10.21977/D92110079

[20] Fancourt D Aufegger L Williamon A. Low-stress and high-stress singing have contrasting effects on glucocorticoid response. Front Psychol. (2015) 6:1242. 10.3389/fpsyg.2015.01242 26388794 PMC4559645

[21] Kreutz G Bongard S Rohrmann S Hodapp V Grebe D. Effects of choir singing or listening on secretory immunoglobulin A, cortisol, and emotional state. J Behav Med. (2004) 27:623–35. 10.1007/s10865-004-0006-9 15669447

[22] Schladt TM Nordmann GC Emilius R Kudielka BM Jong TR de Neumann ID. Choir versus solo singing: effects on mood, and salivary oxytocin and cortisol concentrations. Front Hum Neurosci. (2017) 11:430. 10.3389/fnhum.2017.00430 28959197 PMC5603757

[23] Kreutz G. Does singing facilitate social bonding? Music Med. (2014) 6:51. 10.47513/mmd.v6i2.180

[24] Harris VA Katkin ES Lick JR Habberfield T. Paced respiration as a technique for the modification of autonomic response to stress. Psychophysiol. (1976) 13:386–91. 10.1111/j.1469-8986.1976.tb00850.x 972961

[25] Sakakibara M Hayano J. Effect of slowed respiration on cardiac parasympathetic response to threat. Psychosom Med. (1996) 58:32–7. 10.1097/00006842-199601000-00006 8677286

[26] Whited A Larkin KT Whited M. Effectiveness of emWave biofeedback in improving heart rate variability reactivity to and recovery from stress. Appl Psychophysiol Biofeedback. (2014) 39:75–88. 10.1007/s10484-014-9243-z 24526291

[27] Chin MS Kales SN. Understanding mind-body disciplines: a pilot study of paced breathing and dynamic muscle contraction on autonomic nervous system reactivity. Stress Health. (2019) 35:542–8. 10.1002/smi.2887 31347763 PMC8758201

[28] Steffen PR Austin T DeBarros A Brown T. The impact of resonance frequency breathing on measures of heart rate variability, blood pressure, and mood. Front Public Health. (2017) 5:222. 10.3389/fpubh.2017.00222 28890890 PMC5575449

[29] Bernardi NF Bordino M Bianchi L Bernardi L. Acute fall and long-term rise in oxygen saturation in response to meditation. Psychophysiol. (2017) 54:1951–66. 10.1111/psyp.12972 28840941

[30] Kang J Scholp A Jiang JJ. A review of the physiological effects and mechanisms of singing. J Voice. (2018) 32:390–5. 10.1016/j.jvoice.2017.07.008 28826978

[31] Al'Absi M Everson SA Lovallo WR. Hypertension risk factors and cardiovascular reactivity to mental stress in young men. Int J Psychophysiol. (1995) 20:155–60. 10.1016/0167-8760(95)00029-1 8788218

[32] Dedovic K Duchesne A Andrews J Engert V Pruessner JC. The brain and the stress axis: the neural correlates of cortisol regulation in response to stress. Neuroimage. (2009) 47:864–71. 10.1016/j.neuroimage.2009.05.074 19500680

[33] Krohne HW Egloff B Kohlmann C Tausch A. Untersuchungen mit einer deutschen Version der ‘positive and negative affect schedule' (PANAS). Diagnostica. (1996) 42:139–56.

[34] Tarvainen MP Niskanen J-P Lipponen JA. Ranta-aho PO, Karjalainen PA. Kubios HRV—heart rate variability analysis software. Comput Methods Programs Biomed. (2014) 113:210–20. 10.1016/j.cmpb.2013.07.024 24054542

[35] Laborde S Mosley E Thayer JF. Heart rate variability and cardiac vagal tone in psychophysiological research - recommendations for experiment planning, data analysis, and data reporting. Front Psychol. (2017) 8:213. 10.3389/fpsyg.2017.00213 28265249 PMC5316555

[36] Lehrer PM Vaschillo E Vaschillo B Lu S-E Eckberg DL Edelberg R. Heart rate variability biofeedback increases baroreflex gain and peak expiratory flow. Psychosom Med. (2003) 65:796–805. 10.1097/01.PSY.0000089200.81962.19 14508023

[37] Olsson EM von Scheele B Theorell T. Heart rate variability during choral singing. Music Med. (2013) 5:52–9. 10.1177/1943862112471399

[38] Grape C Sandgren M Hansson L-O Ericson M Theorell T. Does singing promote well-being? An empirical study of professional and amateur singers during a singing lesson. Integr Physiol Behav Sci. (2003) 38:65–74. 10.1007/BF02734261 12814197

[39] Elliot WJ Izzo JL White WB Rosing DR Snyder CS Alter A. Graded blood pressure reduction in hypertensive outpatients associated with use of a device to assist with slow breathing. J Clin Hypertens. (2004) 6:553–9; quiz 560–1. 10.1111/j.1524-6175.2004.03553.x 15470284 PMC8109322

[40] Joseph CN Porta C Casucci G Casiraghi N Maffeis M Rossi M. Slow breathing improves arterial baroreflex sensitivity and decreases blood pressure in essential hypertension. Hypertension. (2005) 46:714–8. 10.1161/01.HYP.0000179581.68566.7d 16129818

